# Causal associations and potential mechanisms between inflammatory skin diseases and IgA nephropathy: a bi-directional Mendelian randomization study

**DOI:** 10.3389/fgene.2024.1402302

**Published:** 2024-07-25

**Authors:** Wenlong Cao, Jing Xiong

**Affiliations:** Department of Nephrology, Union Hospital, Tongji Medical College, Huazhong University of Science and Technology, Wuhan, China

**Keywords:** IgA nephropathy, inflammatory skin diseases, atopic dermatitis, acne, psoriasis, Mendelian randomization

## Abstract

**Background:**

There is growing evidence of an association between inflammatory skin diseases and chronic kidney disease, but the association between inflammatory skin diseases and IgA nephropathy has rarely been studied. Thus, bi-directional Mendelian randomization was employed to explore the causality between inflammatory skin diseases (including atopic dermatitis, acne and psoriasis) and IgA nephropathy.

**Methods:**

The selection of instrumental variables for inflammatory skin diseases and IgA nephropathy were based on genome-wide association studies. Following the heterogeneity and pleiotropy tests, the bidirectional causality was evaluated by inverse variance weighted along with four other approaches. Three atopic dermatitis-related datasets were obtained from the GEO database and then combined. In the combined dataset, the expression of galactose-deficient IgA1-associated genes (including GALNT2, GALNT12, C1GALT1, C1GALT1C1 and ST6GALNAC2) were compared between atopic dermatitis patients and healthy controls.

**Results:**

Atopic dermatitis was associated with an increased risk of IgA nephropathy (OR = 1.054, 95% CI = 1.014–1.095, *p* = 0.007). However, acne and psoriasis showed no significant causal relationship with IgA nephropathy (OR = 0.988, 95% CI = 0.948–1.031, *p* = 0.583; OR = 0.996, 95% CI = 0.966–1.028, *p* = 0.821). In the combined microarray dataset, the expression levels of GALNT12 and C1GALT1C1 in atopic dermatitis patients were significantly lower compared with controls (*p* = 2.3e^−9^; *p* = 0.00067), which may contribute to an increase in aberrant IgA1 synthesis.

**Conclusion:**

Among inflammatory skin diseases, atopic dermatitis was found to increase the risk of IgA nephropathy, which may result from the decrease of GALNT12 and C1GALT1C1 expression and the increase of aberrant IgA1 production. Therefore, active management of atopic dermatitis may help prevent the occurrence and progression of IgA nephropathy.

## Introduction

IgA nephropathy (IgAN) has the highest incidence among primary glomerular diseases. The clinical manifestations of IgAN show high heterogeneity, ranging from asymptomatic gross hematuria to rapid progression of renal function. Despite the existence of multiple treatment strategies for IgAN, approximately 30%–40% of patients will develop renal failure in 20–30 years (Stamellou et al., 2023). The primary pathogenesis of IgAN is overproduction of galactose-deficient IgA1 (Gd-IgA1). Gd-IgA1 and autoantibodies combine to generate an immunological complex that is deposited in the glomerular mesangium, causing inflammation and scarring (Suzuki et al., 2011). Inflammatory skin diseases (ISDs) refer to a class of diseases characterized by the destruction of the skin barrier caused by the disorder of the immune system. Among ISDs, atopic dermatitis (AD), acne and psoriasis are the most common. In recent years, there has been a dramatic increase in the incidence of ISDs, which not only affects the physical and mental health of patients, but also poses a huge economic burden to society (Zhong et al., 2024).

The link between ISDs and chronic kidney disease (CKD) has been demonstrated in studies. In a case-control study of approximately 80,000 participants, patients with ISDs (including AD, psoriasis and hidradenitis suppurative) were found to have an increased risk of developing chronic kidney disease (CKD), but subgroup analysis of the causes of CKD was not carried out in the study (Schonmann et al., 2021). In a recent Mendelian randomization (MR) analysis, AD was also found associated with an increased risk of CKD and subgroup analysis was performed. There were no significant correlations found between AD and any of the etiologies (including diabetic nephropathy, glomerulonephritis, nephrotic syndrome, membranous nephropathy and hypertensive nephropathy), but IgAN was not analyzed in the study (Zhang et al., 2023). At present, studies on the relationship between ISDs and IgAN are so limited that only one cohort study has found a higher risk of IgAN in psoriasis patients (Grewal et al., 2017). Thus, our study aims to investigate the causal associations and potential mechanisms between ISDs (especially AD, acne and psoriasis) and IgAN via MR, which may yield novel insights into the prevention and management of IgAN.

## Materials and methods

### Study design

**FIGURE 1:**
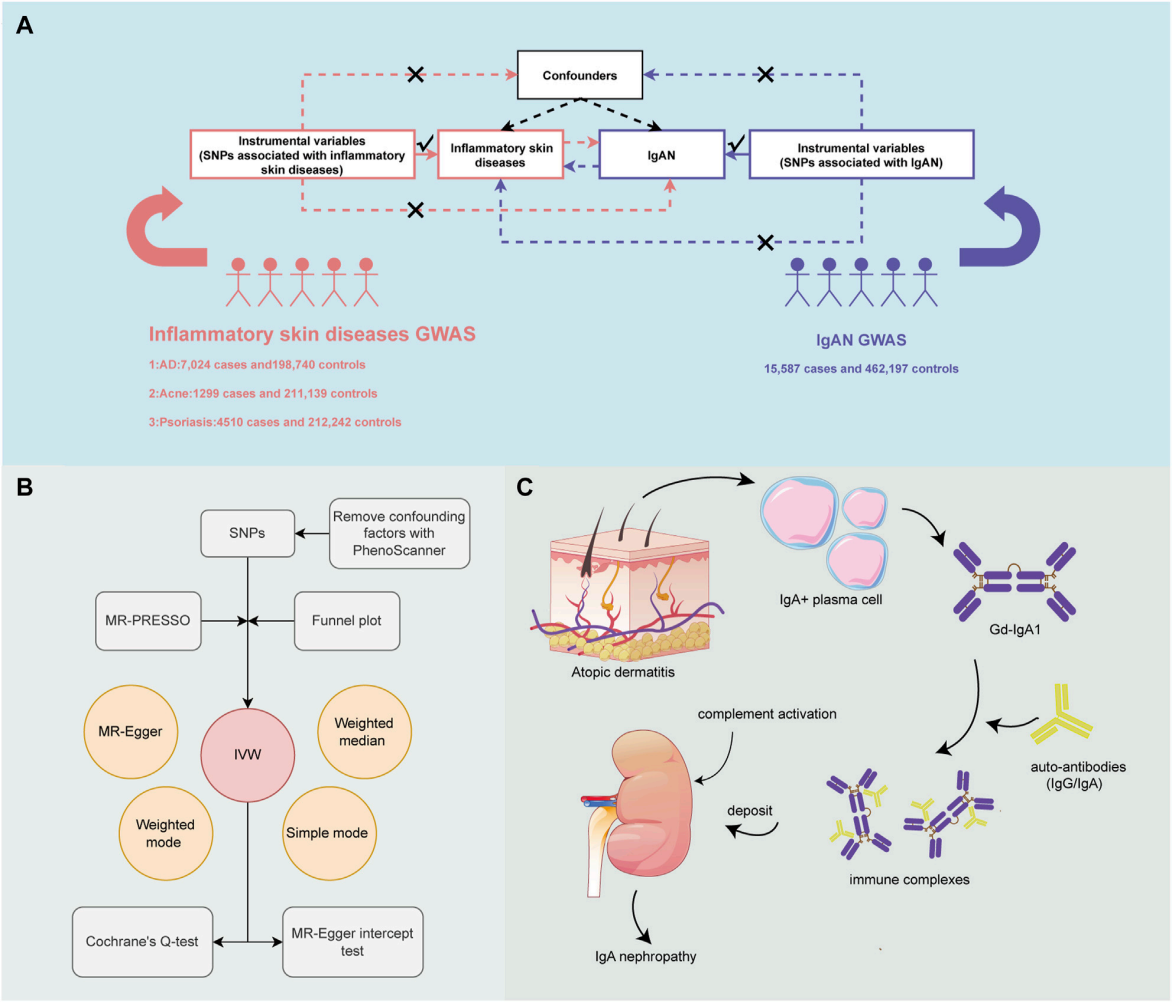
An overview of the study design. **(A)** Three essential assumptions for Mendelian Randomization (MR). **(B)** How the MR analysis was performed step by step. **(C)** Potential mechanism by which atopic dermatitis (AD) increases the risk of IgA nephropathy (IgAN).

In order to evaluate the causality between ISDs and IgAN, a bi-directional two-sample MR study was designed. Three assumptions served as the foundation for MR design: Instrumental variables (IVs) must be strongly correlated with exposure; IVs must be unaffected by any confounding factors; IVs must be connected with outcomes through exposure, rather than through any other causal pathway (Figures 1A, B) (Davey Smith and Hemani, 2014). Next, with bioinformatics methods, the potential mechanism by which AD causes the increased risk of IgAN was explored (Figure 1C).

### Selection of instrumental variables

All of our data was based on independent genome-wide association study (GWAS). IVs of AD, acne and psoriasis came from the FinnGen Consortium while IVs of IgAN came from an available GWAS (Table 1). All of the GWAS data mentioned above can be obtained from IEU GWAS database (https://gwas.mrcieu.ac.uk/). To obtain qualifying IVs, a series of screening steps were performed: IVs were significantly correlated with exposure at the genome-wide level (*p* < 5e^−8^); linkage disequilibrium was removed (r^2^ = 0.001, kb = 10,000) (Clarke et al., 2012; PhenoScanner database was utilized to remove IVs that could cause confounding bias (http://www.phenoscanner.medschl.cam.ac.uk/phenoscanner); palindromic IVs of medium alleles were removed; F statistics were used to exclude weak IVs (F < 10) (Burgess et al., 2011; Palmer et al., 2012).

**TABLE 1:** Descriptions of GWAS data used for analyses.

GWAS ID	Traits	Author	Year	Sample size	Number of SNPS	Population
ebi-a-GCST90018866	IgAN	Sakaue S	2021	4,77,784	24,182,646	European
finn-b-L12_ATOPIC	AD	NA	2021	2,05,764	16,380,443	European
finn-b-L12_ACNE	Acne	NA	2021	2,12,438	16,380,454	European
finn-b-L12_PSORIASIS	Psoriasis	NA	2021	2,16,752	16,380,464	European

### Mendelian randomization analysis

The inverse variance weighted (IVW) model of random effect was used as the primary analysis approach, with the other four methods as supplements, including simple mode, weighted mode, weighted median and MR-Egger (Burgess et al., 2013). To detect and eliminate any outlier which may cause horizontal pleiotropy, MR-PRESSO test was employed (Verbanck et al., 2018). A leave-one–out analysis was performed to ascertain whether there is an association driven by one single nucleotide polymorphism (SNP). Cochran’s Q test and funnel plot were utilized to evaluate heterogeneity, while intercept of MR-Egger was utilized to evaluate pleiotropy (Higgins et al., 2003; Bowden et al., 2015). The causal associations between exposure and outcome were presented with odds ratios and 95% confidence intervals. All statistical analyses were performed in R software (version 4.3.2) with “TwoSampleMR” (Hemani et al., 2018) and “MRPRESSO” packages. The F statistics can be calculated using the following formula: F statistic = beta^2^/se^2^.

### Data preprocessing and merging

Three AD-related microarray datasets including GSE16161 (Guttman-Yassky et al., 2009), GSE32924 (Suárez-Fariñas et al., 2011) and GSE6012 (Olsson et al., 2006), were downloaded from GEO (https://www.ncbi.nlm.nih.gov/geo/) (Table 2). Details of the datasets are presented in the table. The gene expression was standardized with “Limma” package (Ritchie et al., 2015). Then, the three datasets were merged with “sva” package after removing the batch effect.

**TABLE 2:** The microarray datasets information.

Disease	ID	Platform	Tissue	Samples (number)			Experiment type	Author
				Total	Lesion	Normal		
AD	GSE16161	GPL570	Skin biopsies	18	9	9	Array	Guttman-Yassky E
AD	GSE32924	GPL570	Skin biopsies	21	13	8	Array	Suárez-Fariñas M
AD	GSE6012	GPL96	Skin biopsies	20	10	10	Array	Olsson M

### Differential expression of genes related to Gd-IgA1 formation

In the combined dataset, unpaired T-test was conducted to identify the differential expression of five Gd-IgA1 synthesis-related genes between AD patients and controls. The threshold of significance was set as *p* < 0.05. The results were visualized with “grafify,” “ggplot2” and “ggpubr” packages.

## Results

### Characteristics of the selected SNPs

SNPs associated with AD, psoriasis and IgAN were selected according to the predefined standard. Due to the lack of IVs, the *p*-value threshold was suitably expanded for acne SNPs (*p* < 5e^−6^). In the forward MR between psoriasis and IgAN, significant heterogeneity was revealed by Cochrane’s Q test. Thus, after observing the dispersed distribution of funnel plot, we eliminated two outliers (rs138009430 and rs674451) for re-analysis. Two outliers (rs3129962, rs9266216) were detected by MR-PRESSO in the reverse MR analysis between psoriasis and IgAN, and they were also removed. There was no weak IV because the F statistics of all SNPs were larger than 10 (Specific information about the selected SNPs has been included in the Supplementary Material).

### Causal association of ISDs with IgAN via forward MR

**FIGURE 2:**
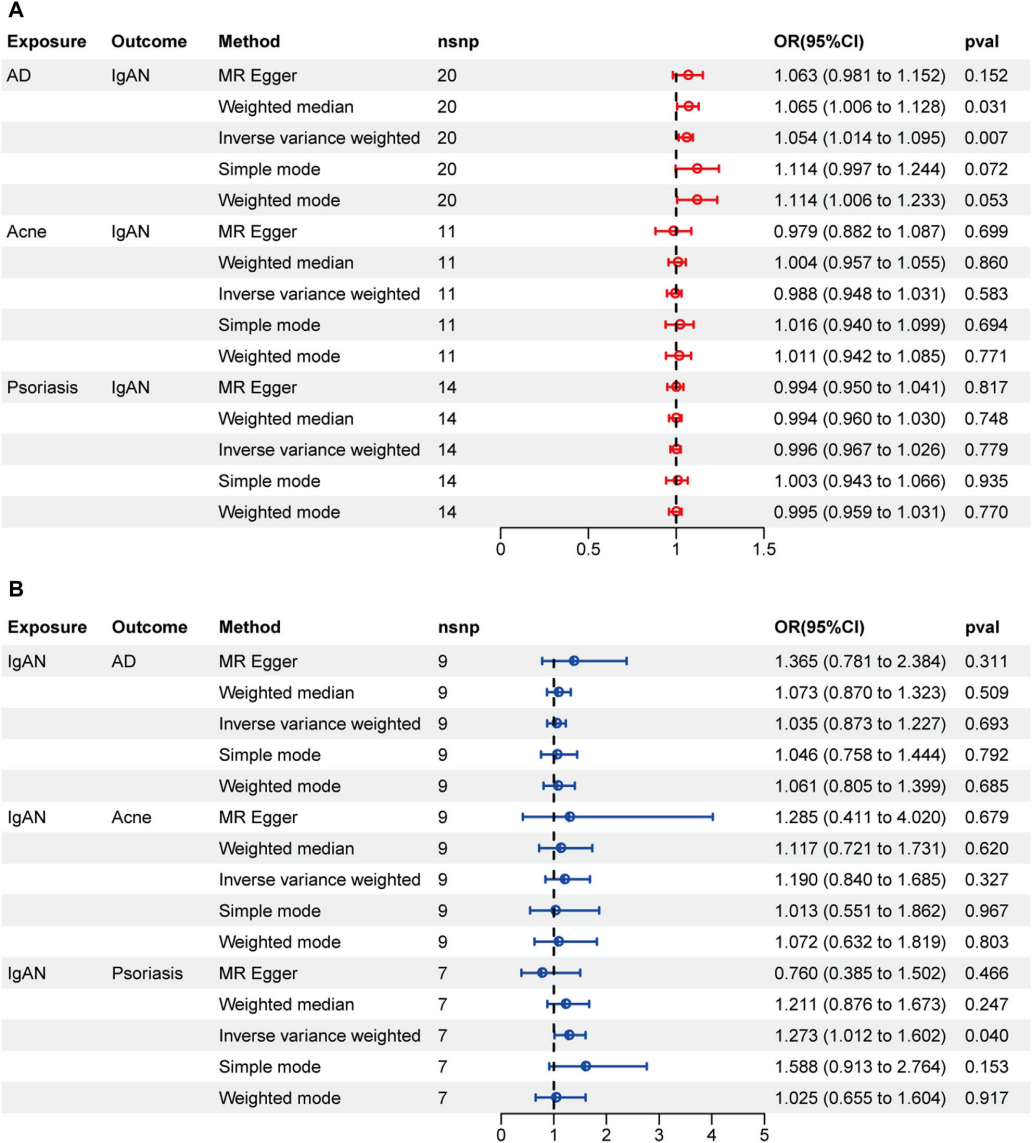
Forest plots of MR results. **(A)** Causal effect of inflammatory skin diseases (ISDs) on IgAN. **(B)** Causal effect of IgAN on ISDs.

A two-sample MR was performed to investigate the causal effect of ISDs on IgAN and the results are displayed in Figure 2A. AD was associated with an increased risk of IgAN (IVW: OR = 1.054, 95% CI = 1.014–1.095, *p*-value = 0.0069). The scatter plot is displayed in Supplementary Figure S1. There was no obvious pleiotropy or heterogeneity, according to the results of the Cochran’s Q test and the MR-Egger regression intercept (Table 3). No single SNP was found to drive the whole effect in leave-one–out analysis (Supplementary Figure S3) and no obvious outliers were found on the funnel plot (Supplementary Figure S4). However, acne and psoriasis showed no significant association with an increased risk of IgAN.

**TABLE 3:** Results of Cochran’s Q test and MR-egger regression intercept.

Exposure	Outcome	Method	Cochran’s Q-derived *P* value	MR-egger intercept-derived *P* value
AD	IgAN	IVW	0.509	0.809
		MR egger	0.447	
Acne	IgAN	IVW	0.122	0.848
		MR egger	0.085	
Psoriasis	IgAN	IVW	0.526	0.942
		MR egger	0.445	
IgAN	AD	IVW	0.302	0.342
		MR egger	0.309	
IgAN	Acne	IVW	0.885	0.893
		MR egger	0.818	
IgAN	Psoriasis	IVW	0.418	0.176
		MR egger	0.613	

### Causal association of IgAN with ISDs via reverse MR

The results of reverse MR are shown in Figure 2B. There was no reverse causality between AD and IgAN (OR = 1.035, 95% CI = 0.873–1.227, *p*-value = 0.693). IVW method indicated that IgAN may serve as a risk factor for psoriasis (OR = 1.273, 95% CI = 1.012–1.602, *p*-value = 0.040), but no significant association was found by the other four methods. Thus, the evidence is insufficient to conclude that IgAN can increase the risk of psoriasis.

### Differential expression of Gd-IgA1 formation-related genes

**FIGURE 3:**
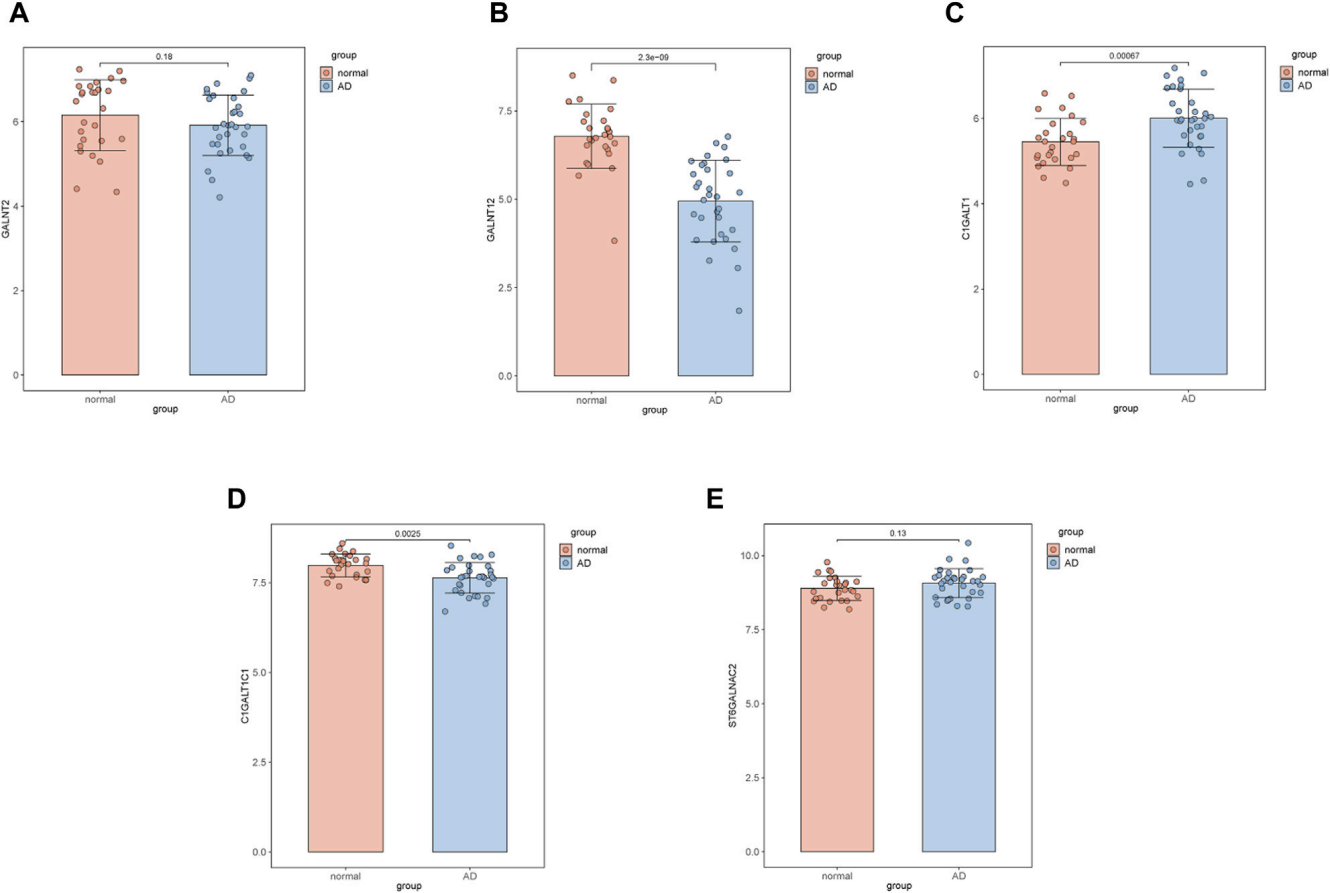
Comparison of the expression of galactose-deficient IgA1-associated genes between AD samples and controls. **(A)** GALNT2. **(B)** GALNT12. **(C)** C1GALT1. **(D)** C1GALT1C1. **(E)** ST6GALNAC2.

Expression products of GALNT2, GALNT12, C1GALT1, C1GALT1C1 and ST6GALNAC2 are involved in Gd-IgA1 formation. Changed expression levels of the genes mentioned above all result in abnormal Gd-IgA1 synthesis. The expression levels of C1GALT1C1 (*p* = 0.0025) and GALNT12 (*p* = 2.3e^−9^) decreased significantly in AD patients, while C1GALT1 increased significantly (*p* = 0.00067). In addition, the decreased GALNT2 expression (*p* = 0.18) and increased ST6GALNAC2 expression (*p* = 0.13) were observed in AD patients, but there was no statistical significance (Figure 3).

## Discussion

Growing evidence has demonstrated that ISDs may be associated with kidney diseases. Y. Schonman discovered that patients with ISDs (including AD, psoriasis and hidradenitis suppurative) were more likely to develop CKD, but the specific mechanism was unknown and the etiology of CKD was not analyzed (Schonmann et al., 2021). Moreover, in the study of H. Zhang, a positive causal relationship was found between AD and CKD via MR analysis. The above association was not observed in subgroup analysis of CKD, which involved diabetic nephropathy, glomerulonephritis, nephrotic syndrome, membranous nephropathy and hypertensive nephropathy, but IgAN was not included in this study (Zhang et al., 2023). It’s widely acknowledged that mucosal immunity plays a key role in the pathogenesis of IgAN while mucosal immune response can be triggered by ISDs. However, studies on the relationship between ISDs and IgAN are very limited.

In epidemiology, randomized controlled study (RCT) is the gold standard for testing scientific hypotheses. However, RCT has certain clinical limitations due to its high cost and long-term follow-up (Fordyce et al., 2015; Harrison, 2016). Despite the convenience and low cost, observational study is susceptible to confounding bias, especially when the sample size is small, which can result in unreliable results (Fewell et al., 2007; Smith et al., 2007). MR is a new epidemiological method to estimate the causal effect of exposure on outcome using GWAS data. The evidence level of MR is second only to RCT (Smith and Ebrahim, 2003). The advantage of MR lies in the ability to avoid the impact of reverse causality and eliminate the interference of confounding factors, increasing the reliability of the results (Davies et al., 2018). Therefore, in this study, the bi-directional MR was chosen to explore the relationship between ISDs and IgAN.

MR can be analyzed through various methods, including IVW, MR-Egger, weighted median, weighted model and simple model. The IVW method is considered as the most accurate method for estimating causality, which combines Wald estimate of each SNP to generate an overall estimate using meta-analysis (Holmes et al., 2017). When judging the causality, results of other methods should also be taken into consideration for comprehensive analysis. Since MR results can be impacted by pleiotropy, MR-PRESSO test is always employed to identify and remove the outliers with horizontal pleiotropy (Verbanck et al., 2018). Pleiotropy can be assessed using the MR-egger regression intercept while heterogeneity can be assessed using the Cochran’s Q test (Higgins et al., 2003; Bowden et al., 2015). All of the methods mentioned above were applied in our research.

A strong positive causal relationship between AD and IgAN was observed in our study, suggesting that AD may act as a risk factor for IgAN. However, acne and psoriasis was not significantly associated with IgAN. It has been revealed in previous studies that ISDs can increase the risk of CKD, but the inner mechanism remains unknown. One plausible explanation is that reactive oxygen species and cytokines can be released into the circulation under the condition of local skin inflammation, ultimately leading to renal damage (Impellizzeri et al., 2014; Eyerich and Eyerich, 2018). In reverse MR, IVW method suggested a statistical association between IgAN and psoriasis, which was not found in the other four methods. Thus, there is no sufficient evidence to confirm the relationship between IgAN and psoriasis, and larger clinical studies may be required to verify it.

The results of MR mentioned above discovered for the first time that AD is a risk factor for the development of IgAN, but the inner pathophysiological mechanism remains unknown. At present, IgAN has been proposed to develop through a “four-hit” process. First, Gd-IgA1 is over-produced and then recognized by autoantibodies. Next, immune complexes form and enter the circulation. They are eventually deposited in the glomerular mesangium, where an inflammatory response is triggered to cause renal damage. It has been revealed that excessive production of Gd-IgA1 in tonsil lymphoid follicles and gut-associated lymphoid tissue may be the source of mesangial IgA deposition (Ambruzs et al., 2014; Hotta et al., 2022). Nevertheless, whether the skin mucosa also produces Gd-IgA1 under pathological circumstances has not yet been reported. It is well known that the gene expression products of GALNT2, GALNT12, C1GALT1, C1GALT1C1 and ST6GALNAC2 are involved in the synthesis of Gd-IgA1. In the initial step of O-glycosylation, N-acetylgalactosaminyltransferase (GalNAc-T) catalyzes the transfer of N-acetylgalactosamine to serine or threonine residues on IgA1. Downregulation of GALNT2 encoding GalNAc-T2 and GALNT12 encoding GalNAc-T12 was found to be linked to aberrant O-glycosylation (Serino et al., 2015; Wang et al., 2021). Galactose on normal IgA1 is transferred to N-acetylgalactosamine residues of IgA1 through core 1 β1, 3 galactosyltransferase (C1β3Gal-T) encoded by C1GALT1. Cosmc, molecular chaperone of C1β3Gal-T, is encoded by C1GALT1C1, which is responsible for the folding and stability of C1β3Gal-T. The function of C1β3Gal-T can be restricted as a result of decreased Cosmc (Li et al., 2007). In addition, Gd-IgA1 may also be associated with increased activity of α2, 6-sialyltransferase II encoded by ST6GALNAC2 since premature sialylation can prevent subsequent galactosylation (Suzuki et al., 2008).

It has been reported in previous research that decreased expression of GALNT2, GALNT12, C1GALT1, C1GALT1C1 and increased expression of ST6GALNAC2 are related to abnormal synthesis of Gd-IgA1. The differential expression of the genes mentioned above between AD samples and controls was analyzed with microarray datasets obtained from GEO. It was demonstrated that the expression levels of C1GALT1C1 and GALNT12 decreased significantly in AD samples. Aberrant expression of the two genes in local lesions of AD patients can lead to abnormal synthesis of Gd-IgA1. Despite the increased expression of C1GALT1, function of galactosyltransferase can still be restricted since C1GALT1C1 was down-regulated, which encodes the molecular chaperone of galactosyltransferase. This may result in galactose unable to be transferred to the N-acetylgalactosamine residues of IgA1. The decreased expression of GALNT12 can lead to the inability of N-acetylgalactosamine to be transferred to serine or threonine residues on IgA1. Therefore, it was hypothesized that AD may affect the glycosylation of IgA1 by down-regulating the expression of C1GALT1C1 and GALNT12, ultimately raising the risk of IgAN.

It has been reported that the inflammatory cascade of AD is mainly mediated by Th2 type immunity, which is accompanied by the production of cytokines, such as IL-4, IL-5, IL-9, IL-13 and IL-31 (Langan et al., 2020). The mRNA expression of Cosmc can be down-regulated by IL-4, causing a decrease in galactosyltransferase activity (Yamada et al., 2010). Th17 may also be involved in the pathogenesis of AD. IL-17 can upregulate proinflammatory and neutrophil-mobilizing cytokines and chemokines. Dysregulated Th17 cell responses mediate a variety of autoimmune and inflammatory diseases, including rheumatoid arthritis, lupus erythematosus, psoriasis, and others (Murdaca et al., 2011). The proportion of Th17 was found to increase in peripheral blood of AD patients and correlated with the severity of the disease (Koga et al., 2008). However, the relationship between Th-17 and Gd-IgA1-related genes has not been reported. Interestingly, miRNAs have been revealed to regulate the expression level of Gd-IgA1-related genes. Overexpression of miR-148b can result in a reduction in C1GALT1, while overexpression of let-7b may lead to a decrease in GALNT2. Let-7b and miR-148b expression showed an obviously positive correlation, indicating the possibility of multiple miRNAs working together to regulate the expression of Gd-IgA1-related genes. More research is needed to determine how the local inflammatory response of AD influences expression of Gd-IgA1-related genes.

Our findings suggested that active management of AD may help prevent the occurrence and development of IgAN. As a calcineurin inhibitor, Cyclosporine A can suppress the immune system through inhibiting the activation of T lymphocytes. Currently, Cyclosporine A has been widely used in various chronic inflammatory diseases, such as systemic sclerosis (Filaci et al., 2001). For AD patients tolerant to conventional treatment, Cyclosporine A can control the disease rapidly and improve the quality of life (Sidbury et al., 2014). For IgAN patients, Cyclosporine A not only regulates the immune system but also reduces proteinuria (Xu et al., 2014). For patients suffering from both AD and IgAN, Cyclosporine A may have better therapeutic effects, which deserves further study.

There are certain limitations in our study. First, all of the GWAS data used for MR was based on European population, and the results were not validated in Asian population. Second, instrumental variables were selected according to the three core assumptions of MR and pleiotropy was evaluated with MR-Egger test. However, it doesn’t mean pleiotropy can be excluded completely. Additionally, bioinformatics methods were employed to investigate the potential mechanism by which AD increases the risk of IgAN. Basic research is required to further confirm our hypothesis.

## Conclusion

In summary, among ISDs, only AD was found to be a risk factor for IgAN. Potential mechanism may be linked to the aberrant expression of Gd-IgA1-related genes. Our findings may provide new insights into the pathogenesis of IgAN and innovative strategies for the prevention and treatment of IgAN.

## Data Availability

The original contributions presented in the study are included in the article/Supplementary Material, further inquiries can be directed to the corresponding author.

## Supplementary material

SUPPLEMENTARY FIGURE S1 Scatter plots visualizing the causality between ISDs and IgAN. **(A)** AD on IgAN. **(B)** acne on IgAN. **(C)** psoriasis on IgAN. **(D)** IgAN on AD. **(E)** IgAN on acne. **(F)** IgAN on psoriasis.

SUPPLEMENTARY FIGURE S2 Forest plots showing the results of Wald ratio method. **(A)** AD on IgAN. **(B)** acne on IgAN. **(C)** psoriasis on IgAN. **(D)** IgAN on AD. **(E)** IgAN on acne. **(F)** IgAN on psoriasis.

SUPPLEMENTARY FIGURE S3 Forest plots showing the results of leave-one–out analysis. **(A)** AD on IgAN. **(B)** acne on IgAN. **(C)** psoriasis on IgAN. **(D)** IgAN on AD. **(E)** IgAN on acne. **(F)** IgAN on psoriasis.

SUPPLEMENTARY FIGURE S4 Funnel plots of single nucleotide polymorphisms. **(A)** AD on IgAN. **(B)** acne on IgAN. **(C)** psoriasis on IgAN. **(D)** IgAN on AD. **(E)** IgAN on acne. **(F)** IgAN on psoriasis.
