# Supplementary figures and images for: Causal associations and potential mechanisms between inflammatory skin diseases and IgA nephropathy: a bi-directional Mendelian randomization study
Source: Front Genet. 2024 Jul 25;15:1402302. doi: 10.3389/fgene.2024.1402302 (PMC11306082; doi:10.3389/fgene.2024.1402302)

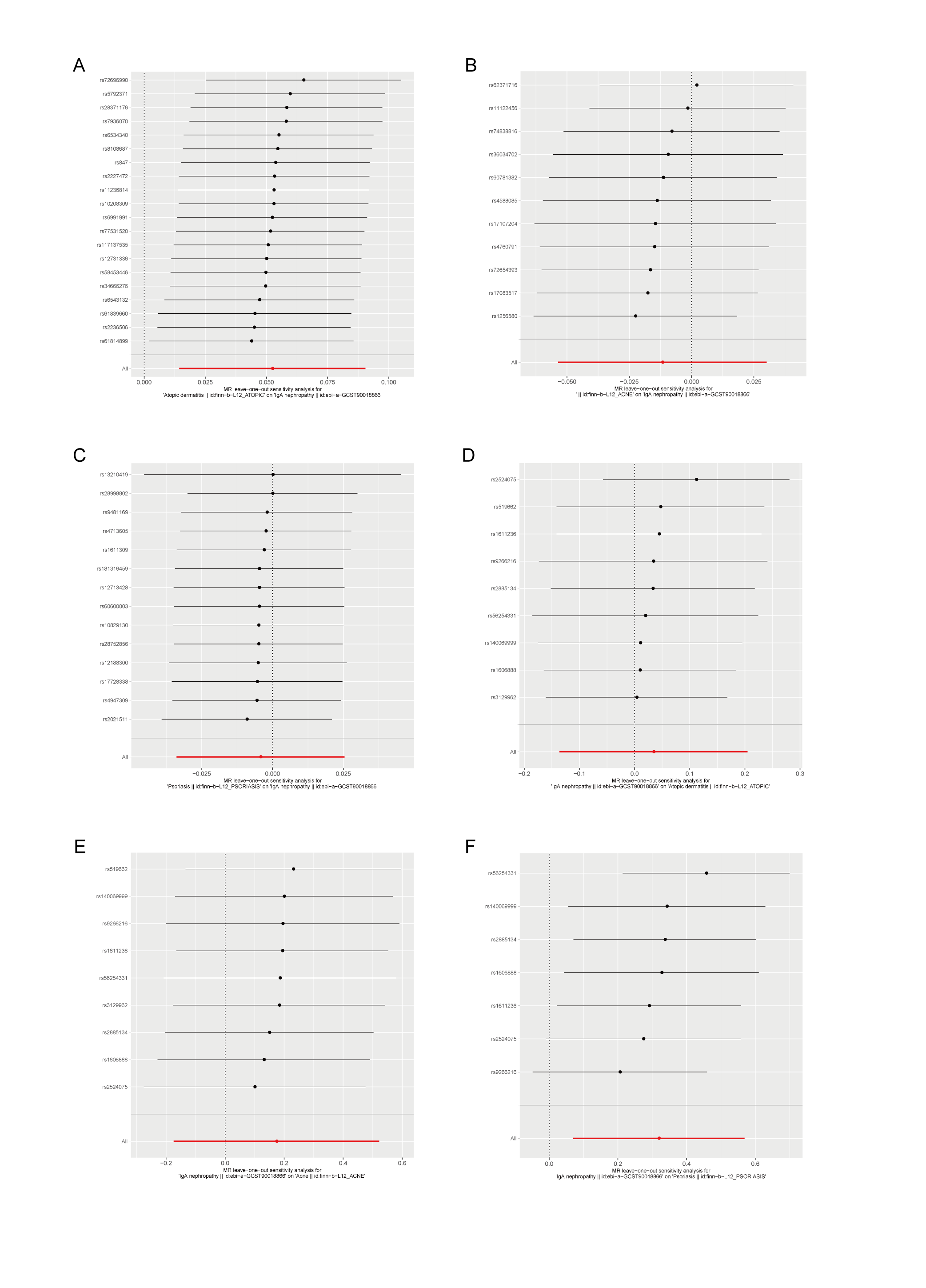

Supplement: Supplementary file 1 [file Image3.TIF]

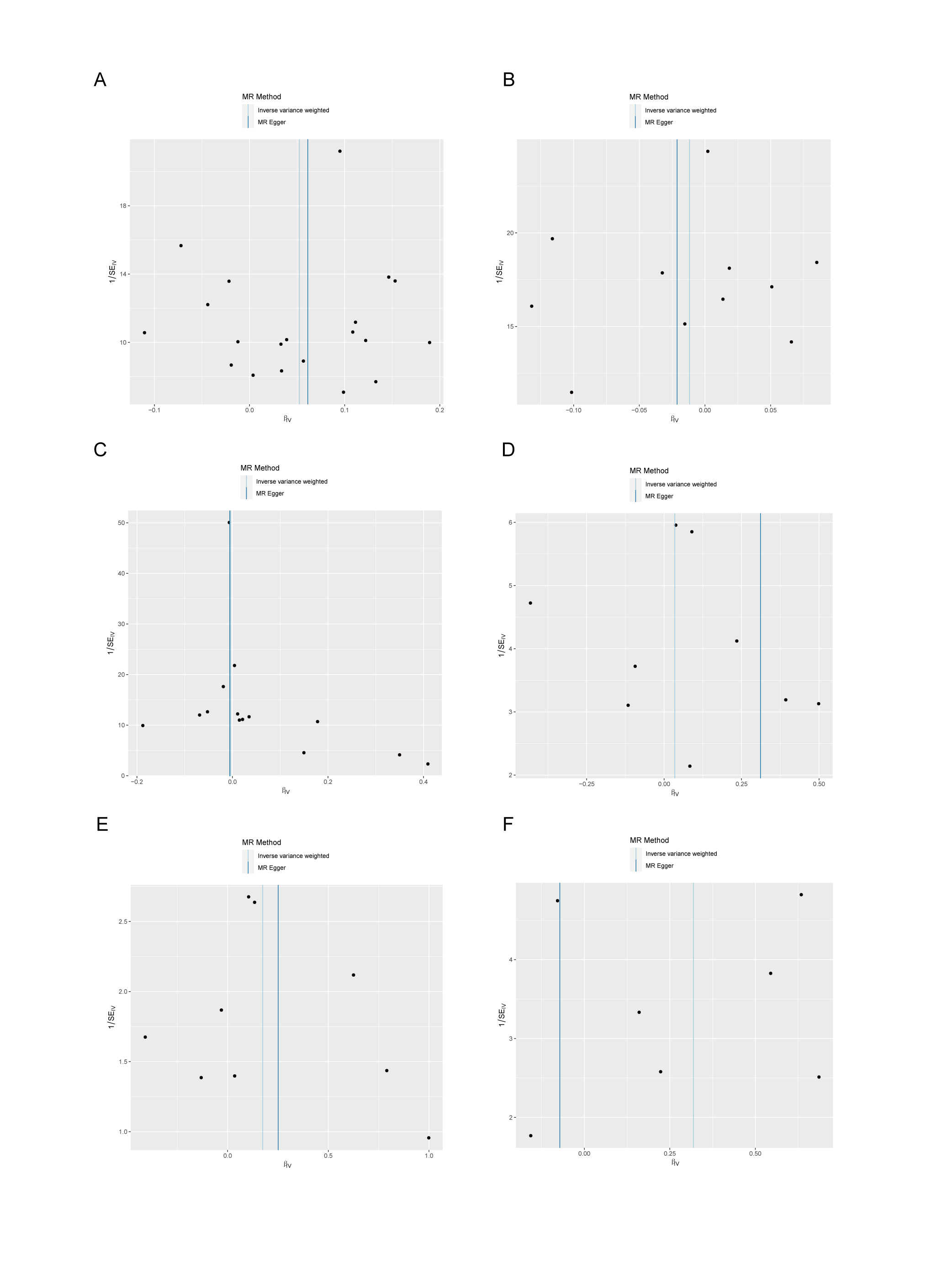

Supplement: Supplementary file 2 [file Image4.TIF]

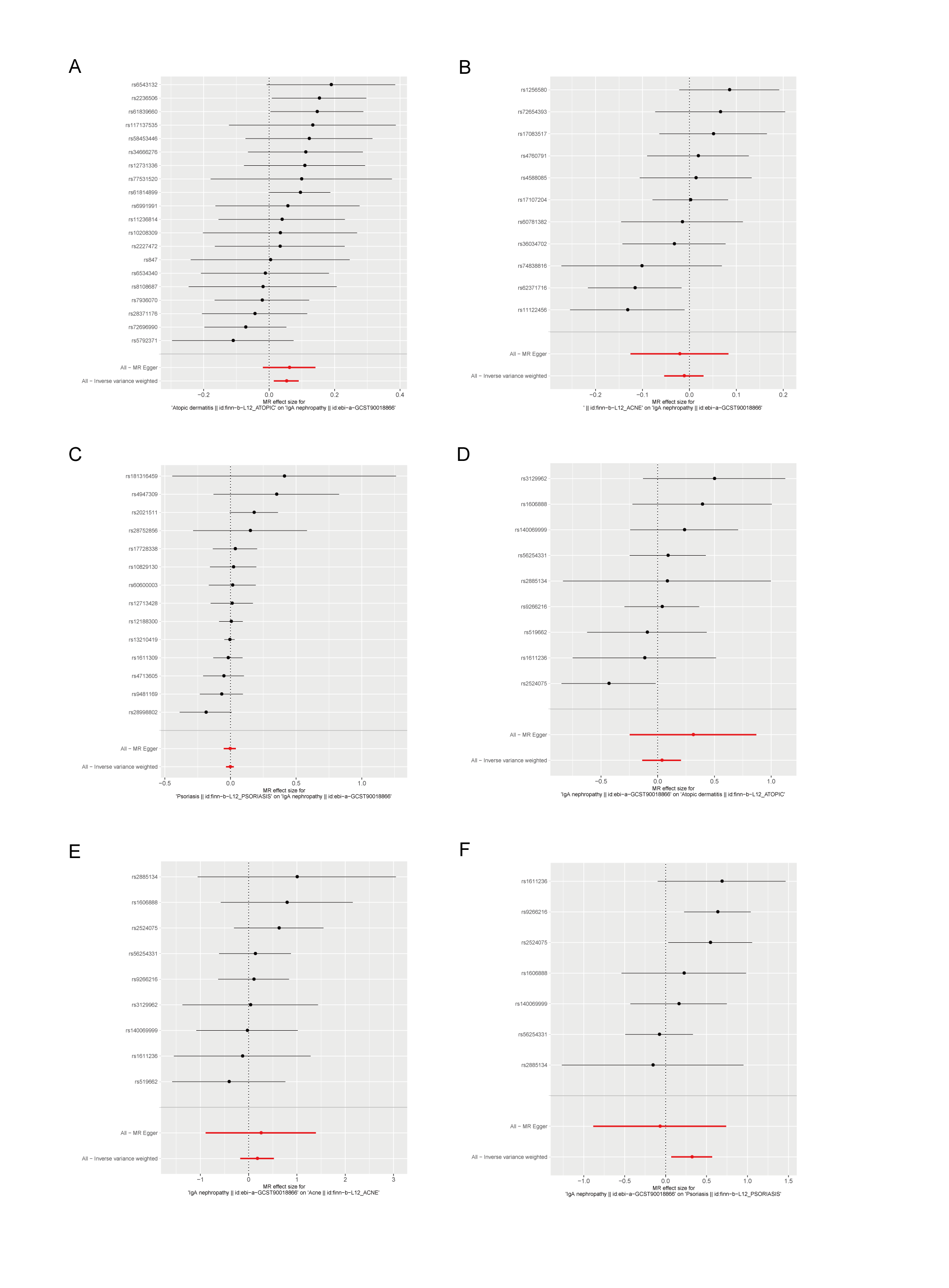

Supplement: Supplementary file 4 [file Image2.TIF]

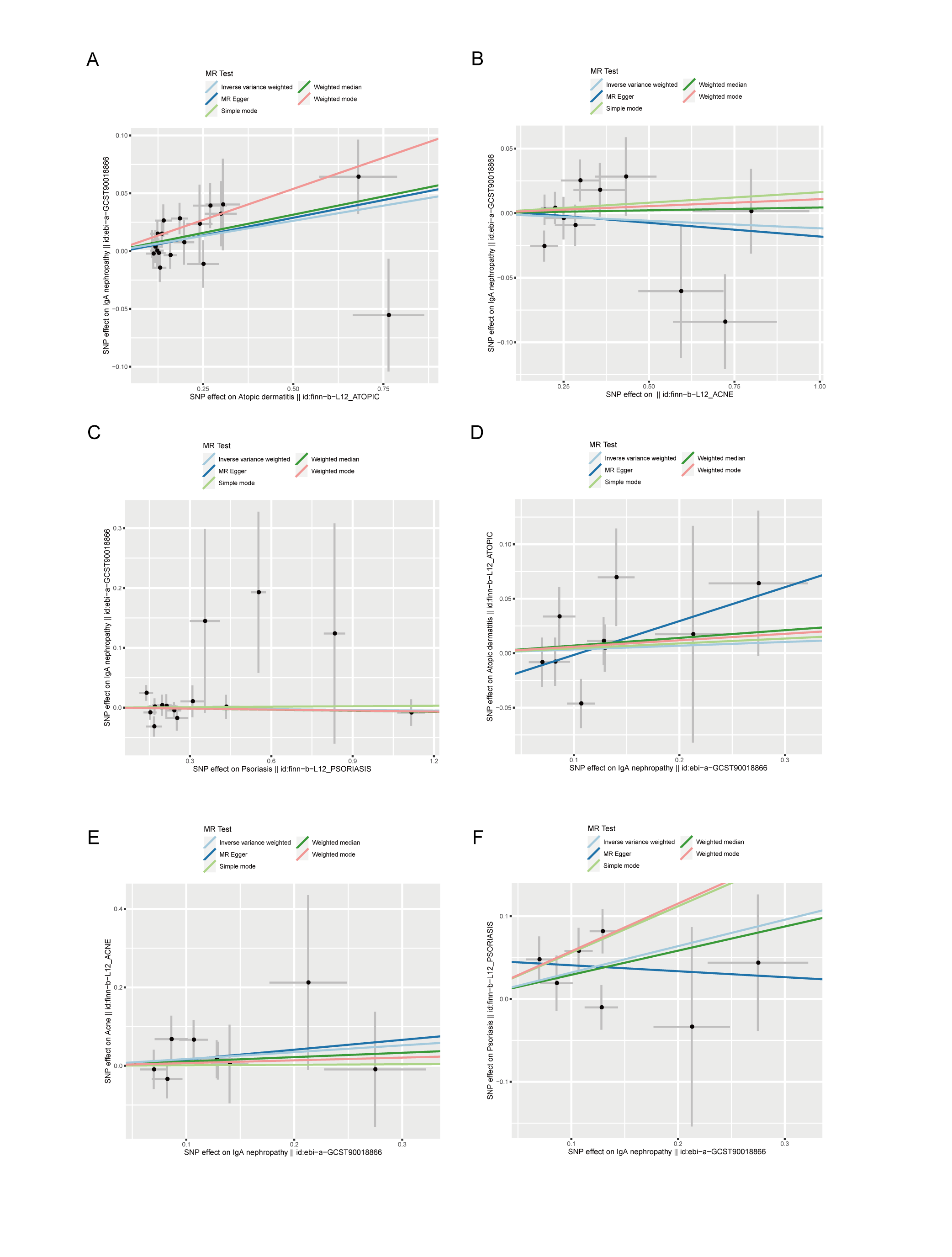

Supplement: Supplementary file 5 [file Image1.TIF]
