# Supplementary material for: Causal associations and potential mechanisms between inflammatory skin diseases and IgA nephropathy: a bi-directional Mendelian randomization study
Source: Front Genet. 2024 Jul 25;15:1402302. doi: 10.3389/fgene.2024.1402302 (PMC11306082; doi:10.3389/fgene.2024.1402302)
Supplement: Supplementary file 3 [file Table2.PDF]

STROBE-MR checklist of recommended items to address in reports of Mendelian randomization studies[1 2]

Item No.	Section		Checklist item	Page No.	Relevant text from manuscript
1	**TITLE and ABSTRACT**		Indicate Mendelian randomization (MR) as the study's design in the title and/or the abstract if that is a main purpose of the study	1	Causal associations and potential mechanisms between inflammatory skin diseases and IgA nephropathy: A bi-directional Mendelian randomization study
	**INTRODUCTION**				
2	**Background**		Explain the scientific background and rationale for the reported study. What is the exposure? Is a potential causal relationship between exposure and outcome plausible? Justify why MR is a helpful method to address the study question	2	The link between inflammatory skin diseases and chronic kidney disease (CKD) has been demonstrated in studies. At present, researches on the relationship between ISDs and IgAN are so limited that only one cohort study has found a higher risk of IgAN in psoriasis patients.
3	**Objectives**		State specific objectives clearly, including pre-specified causal hypotheses (if any). State that MR is a method that, under specific assumptions, intends to estimate causal effects	2	Thus, our study aims to investigate the causal associations and potential mechanisms between ISDs (especially AD, acne and psoriasis) and IgAN via MR, which may yield novel insights into the prevention and management of IgAN.
	**METHODS**				
4	**Study design and data sources**		Present key elements of the study design early in the article. Consider including a table listing sources of data for all phases of the study. For each data source contributing to the analysis, describe the following:		
		a)	Setting: Describe the study design and the underlying population, if possible. Describe the setting, locations, and relevant dates, including periods of recruitment, exposure, follow-up, and data collection, when available.	3	All of our data were based on independent genome-wide association study (GWAS). IVs of AD, acne and psoriasis came from the FinnGen Consortium while IVs of IgAN came from an available GWAS (Table 1). All of the GWAS data mentioned above can be obtained from IEU GWAS database (https://gwas.mrcieu.ac.uk/).
		b)	Participants: Give the eligibility criteria, and the sources and methods of selection of participants. Report the sample size, and whether any power or sample size calculations were carried out prior to the main analysis	3	All of our data were based on independent genome-wide association study (GWAS). IVs of AD, acne and psoriasis came from the FinnGen Consortium while IVs of IgAN came from an available GWAS (Table 1). All of the GWAS data mentioned above can be obtained from IEU GWAS database (https://gwas.mrcieu.ac.uk/).

		c)	Describe measurement, quality control and selection of genetic variants	3	To obtain qualifying IVs, a series of screening steps were performed: (1) IVs were significantly correlated with exposure at the genome-wide level (p<5e-8); (2) linkage disequilibrium was removed (r2=0.001, kb=10000) (8); (3) PhenoScanner database was utilized to remove IVs that could cause confounding bias ; (4) palindromic IVs of medium alleles were removed; (5) F statistics were used to exclude weak IVs (F<10).
		d)	For each exposure, outcome, and other relevant variables, describe methods of assessment and diagnostic criteria for diseases	NA	NA
		e)	Provide details of ethics committee approval and participant informed consent, if relevant	NA	NA
5	**Assumptions**		Explicitly state the three core IV assumptions for the main analysis (relevance, independence and exclusion restriction) as well assumptions for any additional or sensitivity analysis	2	Three assumptions served as the foundation for MR design: (1) Instrumental variables (IVs) must be strongly correlated with exposure; (2) IVs must be unaffected by any confounding factors; (3) IVs must be connected with outcomes through exposure, rather than through any other causal pathway (Figures 1A, B) (7). Next, with bioinformatics methods, the potential mechanism by which AD causes the increased risk of IgAN was explored (Figure 1C).
6	**Statistical methods: main analysis**		Describe statistical methods and statistics used		
		a)	Describe how quantitative variables were handled in the analyses (i.e., scale, units, model)	NA	NA
		b)	Describe how genetic variants were handled in the analyses and, if applicable, how their weights were selected	3	To obtain qualifying IVs, a series of screening steps were performed: (1) IVs were significantly correlated with exposure at the genome-wide level (p<5e-8); (2) linkage disequilibrium was removed (r2=0.001, kb=10000) (8); (3) PhenoScanner database was utilized to remove IVs that could cause confounding bias; (4) palindromic IVs of medium alleles were removed; (5) F statistics were used to exclude weak IVs (F<10).
		c)	Describe the MR estimator (e.g. two-stage least squares, Wald ratio) and related statistics. Detail the included covariates and, in case of two-sample MR, whether the same covariate set was used for adjustment in the two samples	3	The inverse variance weighted (IVW) model of random effect was used as the primary analysis approach, with the other four methods as supplements, including simple mode, weighted

					mode, weighted median and MR-Egger (11).
		d)	Explain how missing data were addressed	NA	NA
		e)	If applicable, indicate how multiple testing was addressed	NA	NA
7	**Assessment of assumptions**		Describe any methods or prior knowledge used to assess the assumptions or justify their validity	3	The threshold of significance was set as $p<0.05$. The causal associations between exposure and outcome were presented with odds ratios and 95% confidence intervals.
8	**Sensitivity analyses and additional analyses**		Describe any sensitivity analyses or additional analyses performed (e.g. comparison of effect estimates from different approaches, independent replication, bias analytic techniques, validation of instruments, simulations)	3	To detect and eliminate any outliers which may cause horizontal pleiotropy, MR-PRESSO test was employed (12). A leave-one–out analysis was performed to ascertain whether there is an association driven by one single nucleotide polymorphism (SNP). Cochran's Q test and funnel plot were utilized to evaluate heterogeneity, while intercept of MR-Egger was utilized to evaluate pleiotropy (13, 14).
9	**Software and pre-registration**				
		a)	Name statistical software and package(s), including version and settings used	3	All statistical analyses were performed in R software (version 4.3.2) with "TwoSampleMR" (15) and "MRPRESSO" packages.
		b)	State whether the study protocol and details were pre-registered (as well as when and where)	NA	NA
	**RESULTS**				
10	**Descriptive data**				
		a)	Report the numbers of individuals at each stage of included studies and reasons for exclusion. Consider use of a flow diagram	4	SNPs associated with AD, psoriasis and IgAN were selected according to the predefined standard. Due to the lack of IVs, the p-value threshold was suitably expanded for acne SNPs ($p< 5e^{-6}$) In the forward MR between psoriasis and IgAN, significant heterogeneity was revealed by Cochrane's Q test. Thus, after observing the dispersed distribution of funnel plot, we eliminated two outliers (rs138009430 and rs674451) for re-analysis. Two outliers (rs3129962, rs9266216) were detected by MR-PRESSO in the reverse MR analysis between psoriasis and IgAN, and they were also removed.

		b)	Report summary statistics for phenotypic exposure(s), outcome(s), and other relevant variables (e.g. means, SDs, proportions)	NA	NA
		c)	If the data sources include meta-analyses of previous studies, provide the assessments of heterogeneity across these studies	NA	NA
		d)	For two-sample MR:<br>i. Provide justification of the similarity of the genetic variant-exposure associations between the exposure and outcome samples<br>ii. Provide information on the number of individuals who overlap between the exposure and outcome studies	NA	NA
11	**Main results**				
		a)	Report the associations between genetic variant and exposure, and between genetic variant and outcome, preferably on an interpretable scale	NA	Supplementary Table 1
		b)	Report MR estimates of the relationship between exposure and outcome, and the measures of uncertainty from the MR analysis, on an interpretable scale, such as odds ratio or relative risk per SD difference	4	AD was associated with an increased risk of IgAN (IVW: OR=1.054, 95%CI=1.014-1.095, p-value=0.0069). There was no reverse causality between AD and IgAN (OR=1.035, 95%CI=0.873 to 1.227, p-value=0.693). IVW method indicated that IgAN may serve as a risk factor for psoriasis (OR=1.273, 95%CI=1.012-1.602, p-value=0.040), but no significant association was found by the other four methods.
		c)	If relevant, consider translating estimates of relative risk into absolute risk for a meaningful time period	NA	NA
		d)	Consider plots to visualize results (e.g. forest plot, scatterplot of associations between genetic variants and outcome versus between genetic variants and exposure)	4	A two-sample MR was performed to investigate the causal effect of ISDs on IgAN and the results are displayed in Figure 2A. The results of reverse MR are shown in Figure 2B.
12	**Assessment of assumptions**				
		a)	Report the assessment of the validity of the assumptions	4	SNPs associated with AD, psoriasis and IgAN were selected according to the predefined standard. Due to the lack of IVs, the p-value threshold was suitably expanded for acne SNPs ($p<5e^{-6}$).
		b)	Report any additional statistics (e.g., assessments of heterogeneity across genetic variants, such as I^2, Q statistic or E-value)	4	There was no obvious pleiotropy or heterogeneity, according to the results of the Cochran's Q test and the MR-Egger regression intercept (Table 2). No single SNP was found to drive the whole effect in

					leave-one–out analysis (Supplementary Figure 3) and no obvious outliers were found on the funnel plot (Supplementary Figure 4).
13	**Sensitivity analyses and additional analyses**				
		a)	Report any sensitivity analyses to assess the robustness of the main results to violations of the assumptions	4	There was no obvious pleiotropy or heterogeneity, according to the results of the Cochran's Q test and the MR-Egger regression intercept (Table 2). No single SNP was found to drive the whole effect in leave-one–out analysis (Supplementary Figure 3) and no obvious outliers were found on the funnel plot (Supplementary Figure 4).
		b)	Report results from other sensitivity analyses or additional analyses	NA	NA
		c)	Report any assessment of direction of causal relationship (e.g., bidirectional MR)	4	A two-sample MR was performed to investigate the causal effect of ISDs on IgAN and the results are displayed in Figure 2A.
		d)	When relevant, report and compare with estimates from non-MR analyses	NA	NA
		e)	Consider additional plots to visualize results (e.g., leave-one-out analyses)	NA	Supplementary Figure 1, Supplementary Figure 2, Supplementary Figure 3, Supplementary Figure 4
	**DISCUSSION**				
14	**Key results**		Summarize key results with reference to study objectives	5	A strong positive causal relationship between AD and IgAN was observed in our study, suggesting that AD may act as a risk factor for IgAN. In reverse MR, IVW method suggested a statistical association between IgAN and psoriasis, which was not found in the other four methods.
15	**Limitations**		Discuss limitations of the study, taking into account the validity of the IV assumptions, other sources of potential bias, and imprecision. Discuss both direction and magnitude of any potential bias and any efforts to address them	6	There are certain limitations in our study. First, all of the GWAS data used for MR is based on European population, and the results were not validated in Asian population. Additionally, bioinformatics methods were employed to investigate the potential mechanism by which AD increases the risk of IgAN. Basic research is required to further confirm our hypothesis.
16	**Interpretation**				

		a)	Meaning: Give a cautious overall interpretation of results in the context of their limitations and in comparison with other studies	6	A strong positive causal relationship between AD and IgAN was observed in our study, suggesting that AD may act as a risk factor for IgAN. Therefore, it was hypothesized that AD may affect the glycosylation of IgA1 by down-regulating the expression of C1GALT1C1 and GALNT12, ultimately raising the risk of IgAN.
		b)	Mechanism: Discuss underlying biological mechanisms that could drive a potential causal relationship between the investigated exposure and the outcome, and whether the gene-environment equivalence assumption is reasonable. Use causal language carefully, clarifying that IV estimates may provide causal effects only under certain assumptions	6	Therefore, it was hypothesized that AD may affect the glycosylation of IgA1 by down-regulating the expression of C1GALT1C1 and GALNT12, ultimately raising the risk of IgAN.
		c)	Clinical relevance: Discuss whether the results have clinical or public policy relevance, and to what extent they inform effect sizes of possible interventions	6	Our findings may provide new insights into the pathogenesis of IgAN and innovative strategies for the prevention and treatment of IgAN.
17	**Generalizability**		Discuss the generalizability of the study results (a) to other populations, (b) across other exposure periods/timings, and (c) across other levels of exposure	NA	NA
	**OTHER INFORMATION**				
18	**Funding**		Describe sources of funding and the role of funders in the present study and, if applicable, sources of funding for the databases and original study or studies on which the present study is based	7	This work was supported by grants from the National Natural Science Foundation of China (No. 81770736). JX provided the financial support and reviewed the manuscript.
19	**Data and data sharing**		Provide the data used to perform all analyses or report where and how the data can be accessed, and reference these sources in the article. Provide the statistical code needed to reproduce the results in the article, or report whether the code is publicly accessible and if so, where	7	The datasets presented in this study can be found in online repositories. The names of the repository/repositories and accession number(s) can be found in the article.
20	**Conflicts of Interest**		All authors should declare all potential conflicts of interest	7	The authors declare that the research was conducted in the absence of any commercial or financial relationships that could be construed as a potential conflict of interest.

This checklist is copyrighted by the Equator Network under the Creative Commons Attribution 3.0 Unported (CC BY 3.0) license.

1. Skrivankova VW, Richmond RC, Woolf BAR, Yarmolinsky J, Davies NM, Swanson SA, et al. Strengthening the Reporting of Observational Studies in Epidemiology using Mendelian Randomization (STROBE-MR) Statement. JAMA. 2021;under review.

2. Skrivankova VW, Richmond RC, Woolf BAR, Davies NM, Swanson SA, VanderWeele TJ, et al. Strengthening the Reporting of Observational Studies in Epidemiology using Mendelian Randomisation (STROBE-MR): Explanation and Elaboration. BMJ. 2021;375:n2233.
